# Docosahexaenoic and Eicosapentaenoic Acid Supplementation Could Attenuate Negative Effects of Maternal Metabolic Syndrome on Liver Lipid Metabolism and Liver Betacellulin Expression in Male and Female Rat Offspring

**DOI:** 10.3390/metabo15010032

**Published:** 2025-01-09

**Authors:** Tomislav Mašek, Petra Roškarić, Sunčica Sertić, Kristina Starčević

**Affiliations:** 1 Department of Animal Nutrition and Dietetics, Faculty of Veterinary Medicine, University of Zagreb, Heinzelova 55, 10000 Zagreb, Croatia; suncicasertic@gmail.com; 2 Department of Chemistry and Biochemistry, Faculty of Veterinary Medicine, University of Zagreb, Heinzelova 55, 10000 Zagreb, Croatia; petra.roskaric@vef.hr (P.R.); kristina.starcevic@vef.unizg.hr (K.S.)

**Keywords:** maternal programming, metabolic syndrome, offspring, lipid metabolism, betacellulin

## Abstract

**Background/Objectives**: This study investigated the effects of maternal metabolic syndrome during pregnancy on hepatic fatty acid metabolism and betacellulin expression in rat offspring. A rat model of maternal metabolic syndrome was created with a high-fructose diet (15% fructose in drinking water for six months). **Methods**: The females with metabolic syndrome were divided into the CON group, the HF group, which received fructose in drinking water, and the HF-DHA group, which received fructose in water and increased amounts of DHA (docosahexaenoic acid) and EPA (eicosapentaenoic acid) in the diet (2.5% fish oil in the diet). The male and female offspring were killed at birth and their liver tissue was analyzed for the fatty acid profile and expression of Δ-9-desaturase and betacellulin. **Results**: When the rat offspring were exposed in utero to maternal fatty acids altered by the high-fructose diet, this resulted in a similarly altered fatty acid profile in the liver, with the most significant changes being Δ-9 desaturation and a dramatic increase in monounsaturated fatty acids. The offspring also showed an overexpression of hepatic betacellulin. Supplementation with DHA and EPA increased the DHA content and normalized the fatty acid composition of oleic acid, saturated fatty acids, linoleic acid and n3-docosapentaenoic acid in the offspring of mothers on a high-fructose diet. In addition, the DHA/EPA supplementation of fructose-fed mothers normalized hepatic Δ-9-desaturase and betacellulin overexpression in the offspring, suggesting that DHA/EPA supplementation affects not only the fatty acid content but also the liver function. **Conclusions**: The changes observed in this study suggest that DHA/EPA supplementation may modulate the effects of maternal programming on disorders of the lipid metabolism in the offspring.

## 1. Introduction

Obesity, metabolic syndrome and type 2 diabetes are now recognized as global health problems. The epidemic proportions of these diseases and the predictions of their increase in the future represent a major challenge for global health care [1]. One of the hallmarks of these pathological conditions is disturbances in lipid metabolism with the characteristic switch to a more pro-inflammatory fatty acid profile and low-grade chronic inflammation. The exact fatty acid profile depends on the dietary model used: excessive fructose consumption vs. a high-fat diet or the combination of both. The most characteristic changes in animal models treated with high levels of fructose in the diet or drinking water are a drastic increase in Δ9-desaturation and a consequent increase in monounsaturated fatty acids (palmitoleic acid, oleic acid, vaccenic acid).

The elongation and desaturation of fatty acids is a highly regulated process in which nutrition is involved: various metabolic products (glucose), hormones (insulin) and the availability of fatty acids in the diet. Other factors also play a role: competition between the n6 and n3 line for rate-limiting enzymes (desaturases), elongases, partitioning into oxidation, various transcription factors (peroxisome proliferator-activated receptor α, PPARα; sterol response element-binding protein-1c, SREBP-1c; liver X receptor, LXR; carbohydrate regulatory element-binding protein, ChREBP; MAX-like factor X, MLX) and microRNA [2,3,4].

Dietary supplementation with docosahexaenoic acid (DHA, C22:6n3) and eicosapentaenoic acid (EPA, C20:5n3) has gained attention following the discovery of the health-promoting effects of these n3 polyunsaturated fatty acids. The complexity of DHA metabolism is related to its complex regulation, particularly of synthesis, which, according to the current model, involves peroxisomal beta-oxidation [5]. The studies on EPA and DHA suggest that they can prevent obesity [6], reduce insulin resistance [7], improve antioxidant defenses [8] and positively influence the inflammatory process [9]. Many other effects of DHA and EPA are currently under investigation with positive or inconclusive effects [10]. The preventive role of DHA and the effect of an increased ratio of n3/n6 fatty acids in the diet on the pathogenesis of metabolic syndrome triggered by excessive fructose consumption has been described in a rodent model.

In addition to the direct influence of the diet on the first generation, there is also evidence that the parental diet can influence the offspring. In this sense, changes in testicular function (the impairment of antioxidant defense, mitochondrial activity and a switch to a pro-inflammatory fatty acid profile) have been observed in fathers who eat a high-fat diet and their offspring for up to two generations [11,12]. The maternal diet could also change the fatty acid profile and cardiac function in female offspring [13]. Therefore, the current study aimed to determine the consequences of a high-fructose diet in the mother on the liver fatty acid profile of the offspring and the degree of Δ-9 desaturation. In addition, we wanted to investigate whether maternal DHA and EPA supplementation can attenuate the disturbances in the lipid metabolism in the offspring of mothers with experimentally induced metabolic syndrome. The expression of betacellulin in the liver was used as a possible marker for the beneficial effects of DHA and EPA.

## 2. Materials and Methods

### 2.1. Animals and Experimental Design

All experimental procedures were performed in accordance with the Croatian Animal Welfare Act and approved by the Croatian National Ethics Committee and the Veterinary Directorate of the Ministry of Agriculture of the Republic of Croatia (approval EP 410/2023).

A rat model of maternal metabolic syndrome was created in which a high-fructose diet (15% fructose in drinking water for six months) was used [14]. The female and male animals in the control group received normal water. The rats with metabolic syndrome were further divided into the HF group, which received only fructose in the drinking water, and the HF-DHA group, which received fructose in the water and increased amounts of DHA and EPA in the diet (2.5% fish oil in the diet, Table 1).

**Figure 1:**
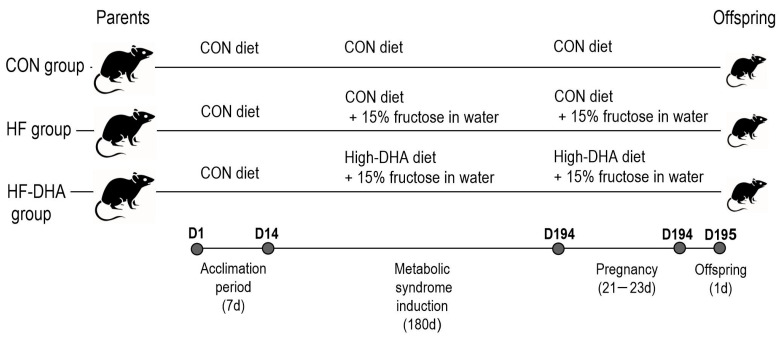
Timeline (days) and feed used during the experiment. CON: control (normal rodent feed); HF: high-fructose (15% fructose in drinking water); and HF-DHA: high-fructose and DHA (15% fructose in drinking water and 2.5% fish oil in the diet).

The parent experiment was performed on 60 Wistar HAN rats in total (30 males, ≈170 g, and 30 females, ≈140 g). The rats were kept in polycarbonate cages and housed under the conditions of a 12 h light–dark cycle at 25 ± 2 °C. All animals had unrestricted access to food and water (tap water). Their body weight was measured daily at the same time (8:00 a.m.) using a digital scale. The schedule and diets used in this study are shown in Figure 1. After the confirmation of metabolic syndrome (significantly increased HOMA-IR), the female and male rats were paired (10 pairs per treatment) and pregnancy was confirmed (vaginal plug, day 1 of gestation). The dietary treatments used in the induction of metabolic syndrome were continued during pregnancy.

### 2.2. Sample Collection

To avoid a possible influence of the litter size, all litters with less than 7 and more than 11 pups were excluded from the experiment. Within 24 h of birth, the offspring were counted, sexed, weighed and euthanized. Livers were isolated, sectioned and stored in an RNA preservative (RNAlater, Thermo Fisher Scientific, Waltham, MA, USA) or frozen at −80 °C for subsequent fatty acid analyses. After the pups, the mothers were also sacrificed using exsanguination under deep anesthesia (Narketan, 80 mg kg^−1^ b.m., and Xylapan, 12 mg kg^−1^ b.m., i.p., Vetoquinol, Bern, Switzerland).

### 2.3. Serum and Liver Tissue Biochemistry

Fasting blood glucose levels were measured using the Accu-Chek Go blood glucose meter [15]. Triglyceride and total cholesterol levels were analyzed using an automated analyzer (SABA-18, Analyser Medical System, Roma, Italy). A commercial rat insulin ELISA kit was used to determine insulin concentrations (Rat Insulin Kit ELISA, Mercodia, Upsalla, Sweden). Insulin sensitivity was measured as HOMA-IR [16] using the following equation:

HOMA IR = (fasting insulin × fasting glucose)/22.5

### 2.4. Quantification of Fatty Acids

Liver lipids were extracted using the Folch method [17] and derivatized using base-catalyzed transmethylation [18]. The extracted lipids were dried under N2 with the addition of BHT as an antioxidant and stored at −80 °C. The fatty acids were transmethylated with 2 M KOH for 15 min at room temperature, extracted in n-hexane and transferred to 1.5 mL vials. The fatty acid profile of the liver tissue was analyzed using GC-MS (QP2010 Ultra, Shimadzu, Kyoto, Japan) with a BPX70 capillary column (0.25 mm inner diameter, 0.25 μm path length, 30 m long, SGE, Austin, TX, USA) under previously described conditions [19]. The injector temperature was set to 250 °C and 1 µL of each sample was injected at a split ratio of 1:80. Helium was used as the carrier gas and the linear velocity was 35 cm/s. The oven program was set as follows: the temperature was set at 40 °C for 3 min, then increased to 130 °C at a rate of 20 °C/min, then increased to 200 °C at a rate of 1.5 °C/min and finally increased to 250 °C at a rate of 45 °C/min and held for 10 °C. The retention times of fatty acid standards (Supelco 37-Component FAME Mix, Merck, Darmstadt, Germany) were used to compare the fatty acid retention times in the samples. The results were expressed as a percentage of the total fatty acids.

### 2.5. Delta 9 Desaturase (SCD1) and Betacellulin Expression

Total RNA was isolated from 30 mg of liver tissue and reverse-transcribed as previously described [20]. The qPCR was performed using a Stratagene MxPro3005 (Agilent Technologies, Santa Clara, CA, USA and Mississauga, ON, Canada) thermal cycler. The oligonucleotides used for quantitative PCR were Δ9D-F, 5′-acattcaatctcgggagaaca-3′; Δ9D-R, 5′-ccatgcagtcgatgaagaac-3′; betacellulin-F, 5′-tctccagtgcgtggtgg-3′; betacellulin-R, 5′-cgagagaagtgggttttcgatt-3′; β-actin-F, 5′-actattggcaacgagcggtt-3′; β-actin-R, 5′-tgtcagcaatgcctgggtac-3′; cyclophilin-F, 5′-cttcttgctggtcttgccattcct-3′; and cyclophilin-R, 5′-ggatggcaagcatgtggtctttg-3′ [14,21]. The gene expression of each sample was analyzed and relatively quantified using the ΔΔCt method (2^ΔΔCt^) after normalization for housekeeping genes [22]. The data were presented as a fold change in the gene expression compared to the control group.

### 2.6. Statistical Analyses

The experimental results obtained were analyzed using the GraphPad Prism 8 program and the data were expressed as mean values ± the SD. The Shapiro–Wilk test was used to test the normality of the data, and a one-way ANOVA, followed by a post hoc Tukey test, was used to compare the treated groups with the control. The standard deviations of the 2^−ΔΔCT^ equation for the tested gene expression were determined according to the established procedure [22]. Significant differences were determined at *p* < 0.05.

## 3. Results

### 3.1. Metabolic Syndrome Induction in Parents

**Figure 2:**
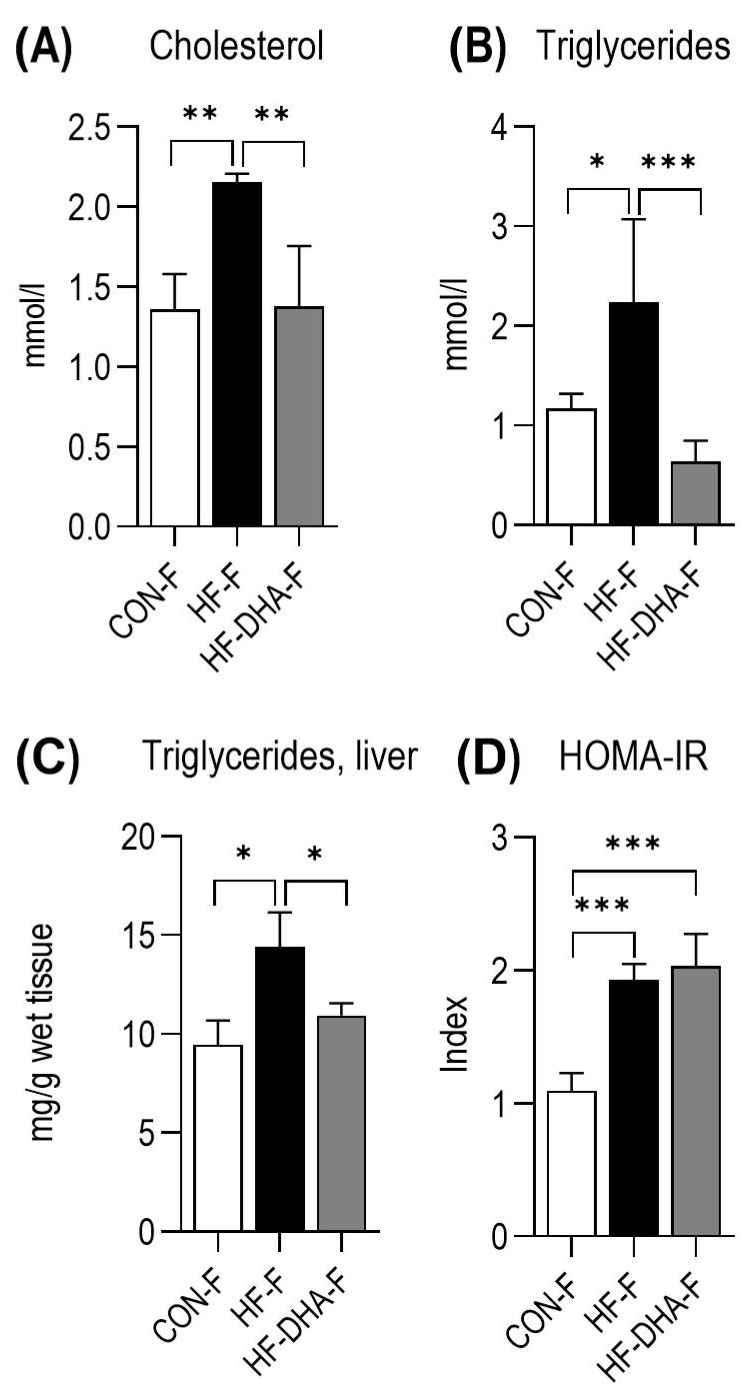
Metabolic characteristics of mothers after 6 months on a high-fructose diet. Serum cholesterol (**A**) and triglyceride levels (**B**). Liver triglyceride content (**C**) and HOMA-IR index (**D**). Values are mean ± SD. * *p* < 0.05, ** *p* < 0.01, *** *p* < 0.001. CON, control diet; HF, high-fructose diet; HF-DHA, high-fructose diet and DHA supplementation; F, female.

The successful induction of metabolic syndrome (after 6 months) was confirmed in the experimental rats (mothers) using the serum biochemistry, which showed increased triglyceride and cholesterol levels in the serum of the HF group (Figure 2A–C). DHA supplementation attenuated these changes, but the HOMA-IR index was still significantly elevated in all HF-treated rats (Figure 2D). 

### 3.2. Saturated and Monounsaturated Fatty Acids and Δ9 Desaturase Expression

**Figure 3:**
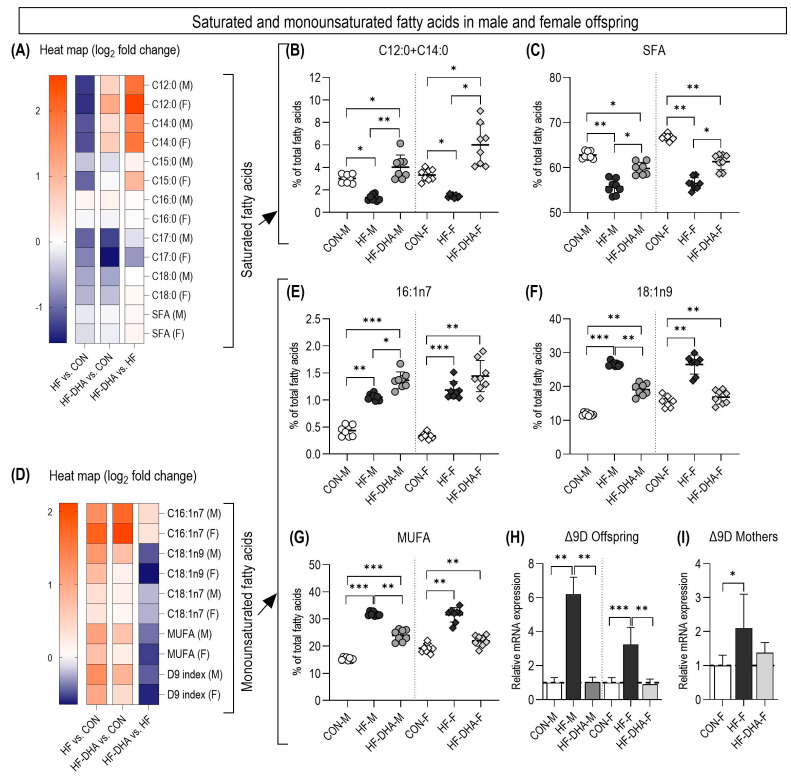
The amount of saturated fatty acids in the liver tissue of the offspring is shown as a heat map (**A**). The most characteristic saturated fatty acids in the liver tissue of offspring were determined through GC-MS and are shown separately: (**B**) the sum of C12:0 and C14:0, (**C**) the summarized profile of the saturated fatty acids. The amount of monounsaturated fatty acids in the liver tissue of the offspring is shown as a heat map (**D**). The most characteristic monounsaturated fatty acids in the liver tissue of the offspring were determined through GC-MS and are shown separately: (**E**) palmitoleic acid (C16:1n7), (**F**) oleic acid (C18:1n9), (**G**) the summary profile of the monounsaturated fatty acids. The expression of the Δ9-desaturase (SCD1) gene was determined through quantitative PCR: (**H**) the expression of Δ9-desaturase in male and female offspring and (**I**) the expression of Δ9-desaturase in the mothers. Values are mean ± SD. * *p* < 0.05, ** *p* < 0.01 and *** *p* < 0.001. CON, control diet; HF, high-fructose diet; HF-DHA, high-fructose diet and DHA supplementation; M, male; F, female.

The offspring of mothers treated with fructose had lower levels of C12:0 and C14:0 fatty acids in the liver, as well as the total SFA content (Figure 3A–C). The addition of DHA/EPA and fructose increased the content of these fatty acids, while the total SFA content was lower compared to the control group and higher compared to the HF group.

Palmitoleic acid, oleic acid and the total MUFA content increased significantly in the offspring of fructose-fed mothers (Figure 3D–G). The combination of DHA/EPA and fructose had different effects on these fatty acids, with the palmitoleic acid content being higher than in the CON and HF groups, while the oleic acid and total MUFA content were intermediate between the CON and HF groups. Quantitative PCR revealed a significant increase in Δ9-desaturase (stearoyl-CoA desaturase-1) gene expression in the liver tissue of high-fructose-treated mothers and their offspring (Figure 3H–I). The addition of DHA/EPA reduced the expression of Δ9-desaturase to the levels of the CON group.

**Figure 4:**
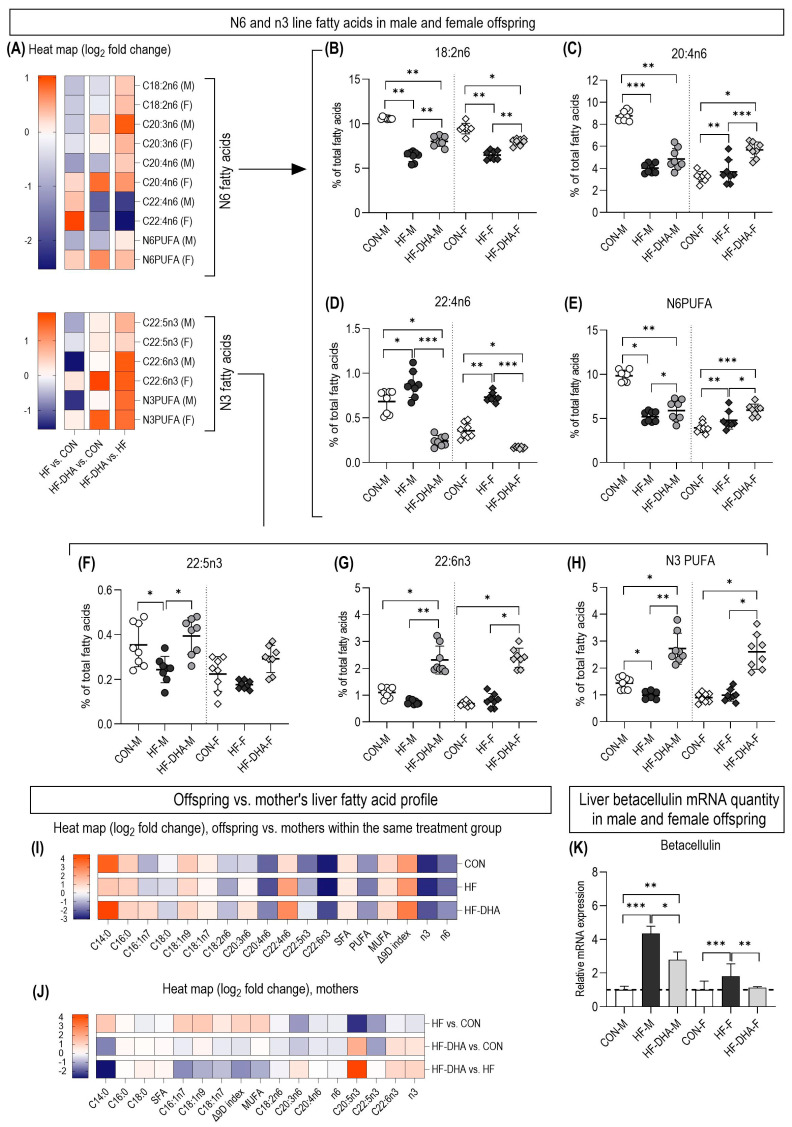
The amount of n6 and n3 polyunsaturated fatty acids (PUFAs) in the liver tissue of the offspring is shown as a heat map (**A**). The most characteristic saturated fatty acids in the liver tissue of the offspring were determined through GC-MS and are shown separately: (**B**) linoleic acid (C18:2n6), (**C**) total n6 PUFAs, (**D**) n6 docosatetraenoic acid (C22:4n6). (**E**) Total n6PUFAs, (**F**) docosapentaenoic acid (C22:5n3), (**G**) docosahexaenoic acid (DHA, C22:6n3) and (**H**) total N3PUFAs. The relation between the changes in the liver fatty acid profile in the mothers and the offspring is presented as a heat map: offspring vs. mothers within the same treatment group (**I**) and changes in the mothers (**J**). The liver expression of the betacellulin gene in the offspring was determined through quantitative PCR (**K**). Values are mean ± SD. * *p* < 0.05, ** *p* < 0.01 and *** *p* < 0.001. CON, control diet; HF, high-fructose diet; HF-DHA, high-fructose diet and DHA supplementation; M, male; F, female.

The excessive consumption of fructose in the parents also altered the amount of n6 fatty acids in the offspring. In general, n6 fatty acids decreased in the HF groups, with the exception of C22:4n6, which increased (Figure 4A–E). The content of n6 fatty acids in the HF-DHA groups also decreased compared to the control group but was significantly higher compared to the HF group. The exception was C22:4n6, whose level was lower compared to the control and HF groups.

The measured n3 fatty acids showed the highest values in the HF-DHA groups (Figure 4F–H). In addition, the values for C22:5n3 and the total n3 fatty acids were lower in the HF group compared to the control group in the male animals.

A comparison of the liver fatty acid profiles showed significant differences between the offspring and the mothers, including lower levels of n3 and n6 polyunsaturated fatty acids, which were similar in all groups (Figure 4I). The most characteristic pathologic changes caused by the excessive consumption of fructose in the mothers were similar in the treated mothers and their offspring, with the increase in all delta 9 desaturation products being the most characteristic (Figure 4J).

Finally, we measured the expression of betacellulin in the liver using quantitative PCR (Figure 4K). The results showed a significant increase in expression in the HF groups. However, DHA/EPA supplementation completely restored the expression to the control levels in both male and female offspring.

## 4. Discussion

Metabolic diseases such as obesity, metabolic syndrome and type 2 diabetes are characterized by changes in lipogenesis in various tissues. Therefore, studying these changes could provide valuable insights into the pathogenesis of these diseases. Nevertheless, the study of fatty acid disorders is challenging due to the highly complex regulation of lipogenesis in the liver and the variety of animal models and dietary interventions used to study these diseases.

To investigate whether a dietary intervention and a developed metabolic syndrome in mothers influence the fatty acid metabolism of their offspring, we performed comprehensive analyses of fatty acids in the liver tissue of the offspring. The liver tissue of the offspring of mothers treated with fructose showed a characteristic pattern of changes in the fatty acid profile. First, we found a reduced content of saturated fatty acids. The content of individual monounsaturated fatty acids was also strongly increased, as was the total MUFA content. In contrast to oleic acid, the content of palmitoleic acid normally has low concentrations in the tissues due to oxidation [23]. Nevertheless, the concentration of palmitoleic acid could greatly increase with a high-carbohydrate diet because it responds immediately to Δ-9 desaturation. Indeed, the expression of Δ-9-desaturase was strongly increased in both the mothers and the offspring, indicating a metabolic response of the offspring to the nutrients they received from the mothers via placental transport. Interestingly, the addition of DHA/EPA to the mothers’ diets had different effects on monounsaturated fatty acids in the offspring’s liver tissue. DHA/EPA attenuated the increase in oleic acid, but palmitoleic acid levels increased even further. As for the expression of Δ-9-desaturase, DHA/EPA supplementation completely restored the expression to the control level, which corresponded to a decrease in the oleic acid content. It should be noted that the content of palmitoleic acid in fish oil, which was the source of DHA/EPA in our study, is high [24]. The fatty acid profile of the maternal diet resembles the fatty acid profile of their milk [25]. Therefore, the high palmitoleic acid content derived from the diet of the mothers in the HF-DHA group was transferred to their offspring, resulting in a high palmitoleic acid content, although DHA reduced Δ-9 desaturation to the normal levels.

An increased content of palmitoleic acid is regularly observed in translational and human studies on obesity/metabolic syndrome/NAFLD when the dietary treatment consists of a high-carbohydrate diet [14,26]. The regular increase in palmitoleic acid in these metabolic diseases and the correlation between the C16:1n-7/C16:0 ratio in serum and liver inflammation, steatohepatosis and fibrosis suggest that this ratio may be a suitable method for the non-invasive diagnosis of non-alcoholic steatohepatitis [27]. It was previously observed that the C16:1n-7/C16:0 ratio was applicable in rats treated with a high-carbohydrate diet, but not in rats treated with a cafeteria or high-fat diet. It should also be noted that a high fish oil intake could also affect this ratio as a marker for the metabolic diseases described.

Previous investigations observed that high-carbohydrate diets decreased the content of essential fatty acids (C18:2n6, C18:3n3) [26,28,29,30,31]. A decrease in the essential linoleic acid was visible in the offspring of fructose-treated mothers, and DHA/EPA supplementation only partially restored values. The changes in PUFAs with more C atoms and double bonds were much less obvious. These discrepancies between studies are likely due to the aforementioned complex regulation of fatty acid synthesis, elongation and desaturation, as well as the different dietary treatments used to induce obesity/metabolic syndrome/NAFLD. The hepatic PUFA profile of the fructose-fed mothers was characterized by a decrease in all n3 and n6 PUFA lines. In the offspring, these changes were only partially visible, suggesting the selective accumulation of fatty acids obtained through placental transport. DHA/EPA supplementation for the mothers restored normal C22:5n3 levels and significantly increased DHA/EPA levels in the offspring, indicating the successful transfer of this health-promoting fatty acid to the offspring.

The health effects of DHA/EPA are well defined in many areas, but the exact molecular mechanisms are still the subject of active research. One of the newly discovered mechanisms is the influence of DHA on betacellulin. Betacellulin is a growth factor that belongs to the epidermal growth factor protein family and has been identified as a potent mitogen for various types of cells [21]. A recent study suggests that the suppression of betacellulin is a mechanism responsible for the anti-inflammatory and anti-fibrotic effects of DHA in non-alcoholic steatohepatitis (NASH), with TGFβ-2 and integrins being the main downstream molecular targets of betacellulin [32]. Our results confirm that hepatic betacellulin was overexpressed in the offspring of mothers with metabolic syndrome. However, the overexpression of betacellulin was attenuated in the offspring exposed in utero to high levels of DHA derived from their mothers. It can be concluded that maternal DHA supplementation has an effect on fatty acid levels (increased DHA) and desaturase expression (decreased Δ9-desasturase expression) in the offspring, but the changes in betacellulin levels also suggest that it may affect liver function in the offspring.

The main finding of our study was that mothers with metabolic syndrome were able to transmit their pathologic liver fatty acid profile and gene expression to their offspring. However, the limitation of our study was that only the first generation was observed. Therefore, we cannot determine whether these effects are short-term or whether they can be observed later in life or even in the F2 generation in the sense of metabolic memory. Therefore, further studies should focus on the lifelong consequences of maternal metabolic syndrome for the offspring and whether these pathological changes in lipid metabolism can be attenuated by dietary supplementation with DHA. Pathologic lipid metabolism is a hallmark of many chronic metabolic diseases. Therefore, betacellulin could play an important role in these future studies and provide valuable insights into the effects of DHA on attenuating these diseases.

## 5. Conclusions

In conclusion, the current study shows that maternal metabolic syndrome can affect lipid metabolism in female and male offspring, with the greatest impact on monounsaturated fatty acid metabolism. This suggests that maternal programming is not only related to the placental transfer of fatty acids to the offspring, but also influences gene expression in the offspring, e.g., fetal liver Δ9 desaturation. The supplementation of mothers with metabolic syndrome with fish oil-derived docosahexaenoic acid had a significant effect on increasing the content of this fatty acid in the fetal liver as well as attenuating the increase in Δ9 desaturation and consequently normalizing the hepatic monounsaturated fatty acid profile. Interestingly, we also found that maternal dietary docosahexaenoic acid affects hepatic betacellulin expression, suggesting an influence on liver pathology. It has already been reported that the influence of fish oil may have a long-lasting effect even after the switch to a normal diet after weaning [25]. Therefore, future research should be extended to the first and second generations to investigate whether the lipid pathology detected at birth could also be present in adulthood and in the second generation, and if so, whether it could be completely or partially attenuated by docosahexaenoic acid supplementation.

## Figures and Tables

**Table 1:** Nutritive composition of diets supplemented to the rats.

	CON	HF	HF-DHA
Crude protein (%)	21.1	21.2	21.1
Crude fat (%)	5.0	5.1	5.3
Crude fiber (%)	4.7	4.7	4.5
Ash (%)	6.2	6.3	6.3
Calcium (%)	1.0	1.1	1.0
Phosphorus (%)	0.8	0.8	0.8
Lysine (%)	1.4	1.4	1.4
Methionine (%)	0.5	0.5	0.5
ME (MJ)	12.7	12.6	12.8
Fatty Acids (% of total fatty acids)			
Palmitic (C16:0)	7.38	7.31	14.80
Palmitoleic (C16:1n7)	nd	nd	5.62
Stearic (C18:0)	2.83	2.29	4.34
Oleic (C18:1n9)	29.67	29.25	29.27
Linoleic (C18:2n6)	52.95	54.18	24.30
Linolenic (C18:3n3)	7.17	6.97	1.02
Eicosapentaenoic (C20:5n3)	nd	nd	9.05
Docosahexaenoic (C22:6n3)	nd	nd	11.59
n6/n3	7.38	7.78	1.12

nd, below quantification level; CON, control diet; HF, high-fructose diet; HF-DHA, high-fructose diet and DHA supplementation.

## Data Availability

The data presented in this study are available on request from the corresponding author.
